# Effectiveness of a community-based intervention (Konga model) to address factors contributing to low viral load suppression among children living with HIV in Tanzania: a preliminary, cluster, randomized clinical trial report

**DOI:** 10.1186/s12889-023-16181-x

**Published:** 2023-07-03

**Authors:** Kihulya Mageda, Khamis Kulemba, Edwin Kilimba, Leornard K. Katalambula, Ntuli Kapologwe, Pammla Petrucka

**Affiliations:** 1 School of Nursing and Public Health, University of Dodoma, PO Box 395, Dodoma, Tanzania; 2 Simiyu Regional Commissioners Office, Bariadi, Tanzania; 3 Amref Health Africa, Dar es Salaam, Tanzania; 4 University of Saskatchewan, Saskatoon, Canada

**Keywords:** Viral load suppression, Antiretroviral therapy, HIV-positive children, Community-based intervention

## Abstract

**Background:**

Despite effective antiretroviral therapy (ART) coverage in other groups living with human immunodeficiency virus (HIV) in Tanzania, virologic suppression among HIV-positive children receiving ART remains unacceptably low. This study evaluated the effectiveness of a community-based intervention (Konga model) in addressing the factor contributing to low viral load suppression among children living with HIV in the Simiyu region, Tanzania.

**Methods:**

This study used a parallel cluster randomized trial. The cluster was only eligible if the health facility provided HIV care and treatment. All eligible resident children aged 2‒14 years who attended the cluster with a viral load > 1,000 cells/mm were enrolled. The intervention included three distinct activities: adherence counseling, psychosocial support, and co-morbidity screening such as tuberculosis. The evaluation was based on patient-centered viral load outcomes measured at baseline and 6 months later. Using a pre- and post-test design, we compared the means of participants in the intervention and control groups. We performed an analysis of covariance. The effect of a Konga was calculated using omega-squared. We used F-tests, with their corresponding p-values, as measures of improvement.

**Results:**

We randomly assigned 45 clusters to the treatment (15) and control (30) groups. We enrolled 82 children with amedian age of 8.8 years(interquartile range(IQR);5.5–11.2), and a baseline median viral load of 13,150 cells/mm (interquartile range (IQR);3600–59,200). After the study, both children in each group had good adherence, with children in the treatment group scoring slightly higher than those in the control group, 40 (97.56%) versus 31(75%61), respectively. At the end of the study, the difference in viral load suppression between the two groups was significant. The median viral load suppression at the end of the study was 50 cells/mm [IQR, (20–125)]. After adjusting for the viral load before the intervention, the effect size of the Konga intervention explained 4% (95% confidence interval [0%, 14.1%]) of the viral load variation at the end of the intervention.

**Conclusion:**

The Konga model demonstrated significant positive effects that improved viral load suppression. We recommend implementing the Konga model trial in other regions to improve the consistency of results.

## Introduction

Human immunodeficiency virus (HIV) is a retroviral virus that attacks the body’s cellular immune system, resulting in acquired immunodeficiency syndrome (AIDS). The attack depletes CD44 cells, making people susceptible to illnesses that a healthy immune system would otherwise prevent [1–3]. However, with the introduction of antiretroviral therapy (ART) and its global accessibility, the negatively affects economic growth ,social impact and mortality has significantly reduced [4, 5]. Thus, HIV infection has become a manageable chronic health condition, enabling people living with this disease to live long and healthy lives [6].

Viral load measurement is used to monitor ART’s efficacy after its initiation, and is regarded as a surrogate marker for disease progression [2, 7]. ART in children aims to suppress HIV replication and halt disease progression while reducing opportunistic infections and morbidities [8, 9].

In children, the primary goal is viral load suppression (VLS) after early ART initiation [10]. Globally, approximately 400,000 children living with HIV under the age of 15 years, who are receiving ART and live in low- and middle-income countries, have not achieved VLS [11]. There has been a tendency for a lower proportion of children, receiving ART, to achieve VLS in East African countries, in comparison to other countries in sub-Saharan Africa [12, 13]. Therefore, there is a high risk of developing AIDS in these areas. In Tanzania, program data and the Tanzania HIV Impact Survey (THIS) have demonstrated that VLS in pediatric patients continues to be low [14–16].

Previous studies have cited factors causing unsuppressed viral loads, including poor drug adherence and co-morbidity [11, 17, 18], malnutrition [19, 20], and underlying tuberculosis (TB) infection [21]. Patients who initially received ART still have advanced diseases [22].

Despite significant ART coverage in other groups living with HIV in Tanzania, the VLS among HIV-positive children receiving ART has remained unacceptably low at 18%[14]. This means 82% of those receiving ART in care and treatment centers (CTCs) have not achieved VLS. Previous studies have demonstrated that enrolling children in ART early, and maintaining good adherence, reduces HIV replication and suppresses the virus [23, 24]. The Tanzanian government has made efforts to improve HIV adherence through the National HIV/AIDS Control Program. Due to the loss of follow-up by caregivers and HIV-exposed infants, these efforts to improve the VLS among children have been ineffective [25]. Thus, UNAIDS [26] recommends the need for sustained engagement and unique contributions from various communities, ranging from small informal groups at the grassroots level to global coalitions.

Several trials in different countries have demonstrated that community-based interventions improve HIV care services for children living with HIV [24, 27–29].

Therefore, we identified the need for a sustainable intervention promoting ART adherence while addressing low VLS among Tanzanian children living with HIV. This study aimed to evaluate the effectiveness of a community-based intervention (Konga model) in addressing the factor contributing to low VLS among Tanzanian children living with HIV.

## Methods and analysis

### Study area

We conducted this study in the Simiyu Region. Administratively, the region comprises six district councils with 218 health facilities (8 hospitals, 17 health centers, and 193 dispensaries), of which 106 sites provide ART services (Tanzania Health Management Information System [HMIS-2020]).

### Study design and population

We used a cluster-randomized trial design with the intervention and control groups running concurrently. Cluster randomization was chosen for practical reasons and to prevent contamination by patient or nurse preferences. The randomization unit was the HIV care and treatment facility, and the unit of analysis was the patient’s viral level. Concerns that the trial’s ability to detect an effect of VLS would be hampered by lower-than-expected children with high viral loads necessitated a protocol amendment 1 month into the study. We approached 25 health centers to join the study and randomly assigned them to either the Konga intervention group (n = 5) or the control arm (n = 20).

### Participants

The cluster was only eligible if the health facility provided HIV care and treatment. Inclusion criteria included children aged 2‒14 years attending a CTC with a viral load > 1,000 cells/mm.

### Recruitment

Healthcare workers (i.e., ART nurses) identified and recruited children aged 2‒14 years with a viral load > 1,000 cells/mm. All caregivers provided written informed consent before participation.

### Intervention

In the intervention group, a study ART nurse identified children with high viral loads, provided adherence counseling sessions and allocated the child to Konga personnel who followed-up with the child at home on a monthly basis. Three interventions were provided: adherence counseling and intensive follow-up (here, the Konga routinely visited the client’s house providing adherence counselling to the child and their caregiver by looking at barriers); follow-up screening for TB and other co-morbidities (members of the Konga visited the children, screened them for opportunistic infections and encouraged them to visit the health facility for further management); and provision of psychosocial support. The control group received the usual routine services.

The National Council of People Living with HIV/AIDS(NACOPHA) is a non-profit, non-governmental organization. It is a national grassroots-based organization of all individuals recognized through organized groups and clusters of people living with HIV (PLHIV) in mainland Tanzania. Since its establishment, NACOPHA has embarked on coordinating the efforts of PLHIV through their district clusters, each known as a “Konga” to address the needs of PLHIV. Hence, we used the PLHIV community to provide services to children.

#### Independent variable

The main dependent variable was whether the child received the intervention or not. The other independent variables were the child’s age, weight, adherence, and opportunistic infection (i.e., TB). The caregivers’ social demographic characteristics, age, sex, level of education, income, and marital status were independent variables that could affect the outcome.

### Outcome measures

We compared the effectiveness of a Konga community using patient-centered interventions aimed at reducing the viral loads of children, in terms of measurements at the start and at the end of 6 months.

### Sample size

A comprehensive explanation of sample size calculations has been reported elsewhere [30], showing all the parameters used in the calculations. Due to the limited number of children, we recalculated the sample size to achieve the same power level to detect a standard effect size of 0.5. We used the analysis of covariance (ANCOVA) method [31] to calculate the required sample size. Assuming that the correlation between the pre-viral load results and post-viral load was 0.6, the mean of group one was 0, that of group 2 was 0.5, the power was 80%, and the standard deviation of both groups was 1. Using the Stata™ function (Sampsi 0.5, power (0.8) st1(1) std2(1) pre (1) r01(0.6)) method (ANCOVA), the sample size was 82 (41 control and 41 treatment).

### Randomization

#### Sequence generation

Health facilities with CTC were classified according to levels, comprising hospitals, health centers, and dispensaries, making three groups. From each group, we randomly selected a health facility (using computer-generated random numbers) to the intervention group and another to the control group.

### Allocation concealment mechanism

A statistician, who was unaware of the study-group assignments, used a table of random numbers for each stratum. Fifteen clusters were randomly allocated to each intervention group and 30 to the usual care group. Either in the intervention or control arm, all children were eligible to be recruited in the control or intervention arm if they were under 15 years of age and had a viral load > 1,000 cells/mm. The allocation remained in the regional medical office until the site had completed the basic introductory training of study personnel (both in standard model and control model clinics [clusters]). After completion of the introductory training, the allocated facilities were delivered directly to the principal investigator.

### Implementation

Forty-five CTC were eligible for the study, and the health authorities agreed to include the clinics in the trial. Informed consent was provided by the caretaker of the child attending the CTC clinic assigned to the Konga model. The child of a caretaker who did not consent was cared for in accordance with the clinic’s usual care. The ART nurse recruited the child and attached to the Konga.

### Blinding

The study was open label, whereby the Konga, caretakers, and children were aware of the interventions received since neither the outcome assessor nor the person receiving the intervention influenced the outcome.

### Statistical methods

During data analyses, we utilised Stata™(Stata Corp LLC, College Station, TX, US) software. For the primary outcome (mean difference in the viral load of HIV-positive children with VLS), we performed ANCOVA using the pre-viral load as a covariate. We compared the mean difference in viral load between the intervention and control groups. Additionally, we used omega-squared to calculate the measure of the effect size. F-tests, with their corresponding p-values, were used as measures of improvement. An interaction test between pre- and post-viral load and age was used to examine the heterogeneity effect. All statistical tests were two-sided; p < 0.05 was considered statistically significant.

## Results

### Participants’ Flow

**Fig. 1:**
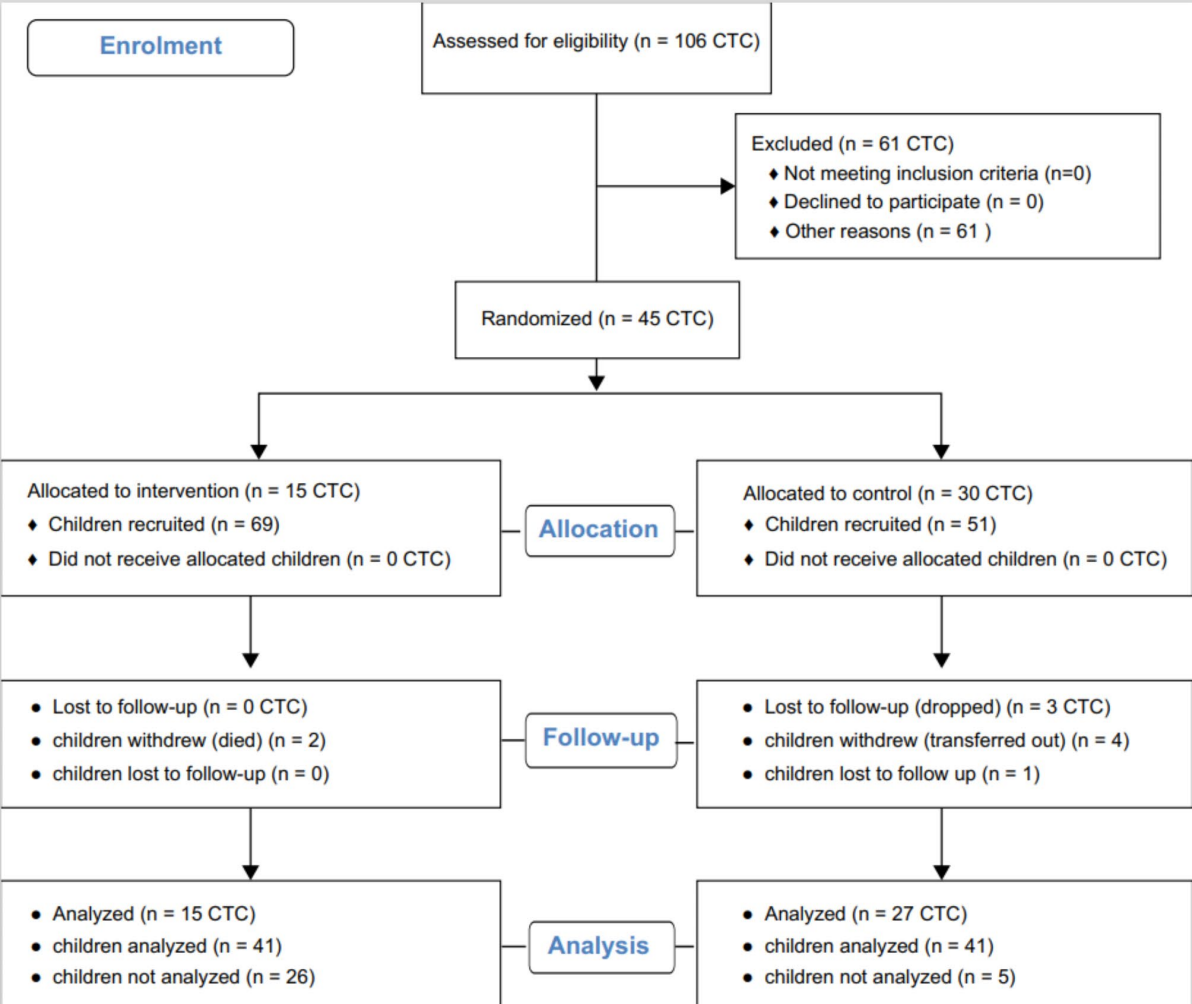
^¥^Flow of clusters and participants from recruitment to analysis ^¥^Adapted from http://www.consort-statement.org/,2010

Figure 1 shows the flow diagram of cluster and child enrollment. We obtained 45 clusters. Of the 45 clusters, 15 were allocated for treatment and 30 were allocated for standard care. All clusters enrolled 69 children for treatment, and 51 for standard care. The children were recruited in July 1, 2022, and the study ended in December 31. We analyzed 82 children at the end of the study.

### Baseline data

Table 1 shows the sociodemographic characteristics of the children and their caregivers. We enrolled 82 children with a median age of 8.8 years ([IQR];5.5–11.2) in the two groups and a baseline median viral load of 13,150 (interquartile range [IQR], 3600–59,200). Most of the caregivers were peasants, with only 48 (59.26%) who had completed primary school. After the study, both children in each group achieved acceptable good adherence with children in the treatment group achieving higher adherence than those in the control group (40 (97.56%) versus 31 (75.61%), respectively). One case of a child infected with TB was identified in the treatment group.

**Table 1:** Sociodemographic characteristics of children and caregivers before and after the intervention, N = 82

Variable	Control	Intervention	Total	P-values
Median age(child)years [IQR]	8.9[5,4–11’1]	8.7[6–12]	8.8 [5.5–11.2]	
Median pre-viral load [copies/mL, IQR]	12,195[6170–29,300]	17,400[3600–73,771]	13,150[3600–59,200]	
Median post-viral load [copies/mL, IQR]	65 [43–200]	20[11–64]	50 [20–125]	
**Distance to the facility, n (%)**				
Living near (< 10 km)	29 (78.38)	27(67.50)	56 (72.73)	0.643
Living far (> 10 km)	8 (21.62)	13 (32.50)	21 (27.27)	
**Sex, n (%)**				1
Female	19 (46.34)	19 (46.34)	38 (46.34)	
Male	22 (53.66)	22 (53.66)	44 (53.66)	
**Relationship, n (%)**				
Both parents	4 (9.76)	11 (26.83)	15 (18.29)	0.243
Father only	8 (19.51)	6 (14.63)	14 (17.07)	
Grandparent	5 (12.20)	7 (17.07)	12 (14,63)	
Mother only	19 (46.34)	12 (29.27)	31 (37.80)	
Relative	5 (12.20)	5 (12.20)	10 (12.20)	
**Education level (caregivers), n (%)**				
None	17 (41.46)	14 (35.00)	31 (38.27)	0.821
Primary	23 (56.10)	25 (62.50)	48 (59.26)	
Secondary	1 (2.44)	1 (2.50)	2 (2.47)	
**Source of income caregiver), n (%)**				
Petty business	3 (7.32)	1 (2.44)	4 (4.88)	0.616
Employed	0	1 (2.44)	1 (1.22)	
Peasant	38 (92.68)	39 (95.12)	77 (93.90)	
**Opportunistic infection (TB), n (%)**				
No	40 (100)	41 (97.56)	81 (98.78)	0.500
Yes	0	1 (2.44)	1 (1.22)	
**Pre-adherence Status, n (%)**				
Good	0	1 (2.44)	1 (1.22)	0.500
Poor	41 (100)	40 (97.56)	81 (98.78)	
**Post-adherence status, n (%)**				
Good	31 (75.61)	40 (97.56)	71 (86.59)	0.007
Poor	10(24.39)	1(2.44)	11(13.41)	

**Table 2:** The fitted model of the difference in viral load suppression results between the control and the intervention group after the study

Sources	Partial SS	Df	MS	F	Prob > F
Model	40,620,624	2	20,310,312	34.48	0.0001
Konga	2532703.4	1	2532703.4	4.30	
Pre-viral load	38,497,999	1	38,497,999	65.35	0.0414
Residual	46,536,739	79	589072.64		0.0001
Total	87,157,362	81	1076016.8		

Table 2 presents a comparison of the results of the viral load suppression between the two groups after the study. After accounting for the viral load measurements taken after the intervention began, the difference in viral load suppression between the two groups (at the end of the study) was significant [F (1,79) = 4.3, p = 0.0414]. Based on these p-values, we can reject the overall null hypothesis of the equality of the means of viral load suppression between the groups.

**Table 3:** The examination of the difference of the adjusted means for the viral load after intervention

Variable	Delta-method		95% Confidence interval	
Konga	Margins	Std. error		
Control	516.7021	119.8792	278.0885	755.3156
Treat	165.1272	119.8792	-73.48638	403.7408

Table 3 presents a comparison of the difference in the adjusted means of the viral load in the intervention and control groups after the study. After we accounted for viral load at the beginning, the mean viral load for the control condition at the end of the study was 516.7 compared to 156.1 for the Konga intervention; thus, there was a greater reduction in viral load in the treatment group than in the control group.

**Table 4:** The measure of the effect size of Konga in viral load suppression

Source	Omega-squared	df	90% Confidence interval	
Model	0.4525433	2	0.3047639	0.5480826
Konga	0.0396098	1	0	0.1360604
Pre-viral load	0.4458052	1	0.3066878	0.547704

Table 4 shows the omega-squared values for the overall model (0.4525). After adjusting for the viral load before the intervention the effect of the Konga intervention explains 4% of the variance in viral load suppression at the end of the intervention.

### Harm

In the treatment and control groups, there was no evidence of any unintended harm during and after the intervention.

## Discussion

This Konga model study was Tanzania’s first cluster-randomized clinical control trial study that used the community as a foundation to address challenges in viral load suppression. The existing community of People Living with HIV (Konga) enhanced ART adherence for VLS in children.

This study showed that community-based interventions (Konga) affect viral load suppression in children. The findings indicate that the Konga model improves medication adherence and subsequent viral load suppression in children receiving HIV treatment and care. The Konga personnel (who are also living with HIV) reduce stigma and provide psychosocial support to caregivers and their corresponding children living with HIV. Ibrahim & Sidani [32] reported that community-based interventions highlighted the importance of carefully designing organization-based HIV prevention interventions in a way that improves their effectiveness and efficiency. Thus, in this study, we carefully designed a Konga model of community participation to promote retention and adherence to treatment, focusing on home-based follow-up and psychosocial and peer support, similar to the recommendation by Mukherjee et al. [26]. Previous studies have demonstrated that poor adherence and retention in ART care detract from children living with HIV [33–36]. In this intervention, we used Konga to promote retention and adherence to ART among children receiving ART to reduce their viral loads.

Although our study identified only one case of TB, the Konga model can increase TB detection during HIV assessments. Opportunistic infections, including TB, have been shown to hamper VLS in children receiving ART [11, 17, 18, 37]. Another study demonstrated that ART initiation in children reduced the incidence of TB [22]. In the control group, the Konga model conducted active home visits to screen children for TB and other co-morbidities. Anígilájé et al. reported that screening helped to identify early infection and facilitated referral to a treatment point [22].

### Strength and limitations

Using pre-viral load as a covariate was an excellent way of increasing the power of the study. However, the observed intervention effect would probably have been more significant if the trial had not been conducted in a single region to prevent spillover to the control group.

Our analysis was tested using different means before and after the study, indicating that the distribution of viral load was positively skewed. This may have influenced the results.

#### Generalizability

Although our trial was a success from a methodological and practical standpoint, our results are generalizable. As the sample size of 82 was small, extensive analysis was impossible. Future randomization should include more extensive samples of children receiving Konga, and measurement of clinic travel distances and other clinically important covariates that affect external validity need to be considered.

### Interpretation

The Konga model is effective in viral load suppression in children living with HIV and enrolled in care and treatment clinic. The Konga model enhanced adherence by addressing ART non-adherence factors among this population throught continuity with adherence counseling, psychosocial support, and TB screening at home.

We recommend that the Konga model trial be replicated in other regions to ensure consistency of results; after that, it can be enrolled and used elsewhere.

t.

## Data Availability

The datasets generated and/or analyzed during the current study are available in the [fig share] repository[ 10.6084/m9.figshare.22141400.v2 ].

## Abbreviations

AIDS: Acquired immunodeficiency syndrome
ANCOVA: Analysis of covariance
ART: Antiretroviral therapy
CI: Confidence interval
CTCs: Care and treatment centers
HIV: Human immunodeficiency virus
IQR: Interquartile range
PLHIV: People living with HIV
TB: Tuberculosis
THIS: Tanzania HIV impact survey
VLS: Viral load suppression
